# Influence of eye movement on lens dose and optic nerve target coverage during craniospinal irradiation

**DOI:** 10.1016/j.ctro.2021.08.009

**Published:** 2021-08-29

**Authors:** Bianca A.W. Hoeben, Enrica Seravalli, Amber M.L. Wood, Mirjam Bosman, Witold P. Matysiak, John H. Maduro, Astrid L.H.M.W. van Lier, Matteo Maspero, Gijsbert H. Bol, Geert O. Janssens

**Affiliations:** a Department of Radiation Oncology, University Medical Center Utrecht, Utrecht, the Netherlands; b Princess Máxima Center for Pediatric Oncology, Utrecht, the Netherlands; c Radboud University, Nijmegen, the Netherlands; d Department of Radiation Oncology, University of Groningen, University Medical Center Groningen, Groningen, the Netherlands

**Keywords:** COM, center of mass, CSI, craniospinal irradiation, CTV_voxelwise min_, voxelwise minimum CTV, D98_OON_, D98 orbital optic nerve, ITV_optic disc_, internal target volume around optic discs, MLD, mean lens dose, OON, orbital optic nerve, PBS, pencil-beam scanning, PRV_lens_, planning organ-at-risk volume around lenses, sCT, synthetic CT, SIOPE, European International Society for Paediatric Oncology, Craniospinal irradiation, VMAT, 3D-conventional, Proton, Lens, Optic nerve

## Abstract

**Purpose:**

Optic nerves are part of the craniospinal irradiation (CSI) target volume. Modern radiotherapy techniques achieve highly conformal target doses while avoiding organs-at-risk such as the lens. The magnitude of eye movement and its influence on CSI target- and avoidance volumes are unclear. We aimed to evaluate the movement-range of lenses and optic nerves and its influence on dose distribution of several planning techniques.

**Methods:**

Ten volunteers underwent MRI scans in various gaze directions (neutral, left, right, cranial, caudal). Lenses, orbital optic nerves, optic discs and CSI target volumes were delineated. 36-Gy cranial irradiation plans were constructed on synthetic CT images in neutral gaze, with Volumetric Modulated Arc Therapy, pencil-beam scanning proton therapy, and 3D-conventional photons. Movement-amplitudes of lenses and optic discs were analyzed, and influence of gaze direction on lens and orbital optic nerve dose distribution.

**Results:**

Mean eye structures’ shift from neutral position was greatest in caudal gaze; −5.8±1.2 mm (±SD) for lenses and 7.0±2.0 mm for optic discs. In 3D-conventional plans, caudal gaze decreased Mean Lens Dose (MLD). In VMAT and proton plans, eye movements mainly increased MLD and diminished D98 orbital optic nerve (D98_OON_) coverage; mean MLD increased up to 5.5 Gy [total ΔMLD range −8.1 to 10.0 Gy], and mean D98_OON_ decreased up to 3.3 Gy [total ΔD98_OON_ range −13.6 to 1.2 Gy]. VMAT plans optimized for optic disc Internal Target Volume and lens Planning organ-at-Risk Volume resulted in higher MLD over gaze directions. D98_OON_ became ≥95% of prescribed dose over 95/100 evaluated gaze directions, while all-gaze bilateral D98_OON_ significantly changed in 1 of 10 volunteers.

**Conclusion:**

With modern CSI techniques, eye movements result in higher lens doses and a mean detriment for orbital optic nerve dose coverage of <10% of prescribed dose.

## Highlights

• While optic nerves are part of the CSI target volume, lenses need to be spared.
• Lens and optic disc movement for different gaze directions was evaluated in MRI scans.
• Eye movement influence on lens and optic nerve CSI dose distribution was analyzed.
• With modern radiotherapy techniques, any eye movement increases the mean lens dose.
• Maximum eye movements decrease mean orbital optic nerve D98 <10% of prescribed dose.

## 1 Introduction

Patients with medulloblastomas or other pediatric tumors with leptomeningeal spread, such as germ-cell tumors, atypical teratoid rhabdoid tumors, and ependymomas, have an indication for craniospinal irradiation (CSI) [1]. Recently, the European International Society for Paediatric Oncology (SIOPE) Brain Tumor Group consensus on craniospinal target volume delineation was published, which is applicable for advanced radiotherapy techniques [1]. Inclusion of the full length of both optic nerves, with the optic discs, is recommended in the guideline. Meanwhile, MRI data illustrated cerebrospinal fluid (CSF) extension into the dural sheath of the optic nerves up to the posterior part of the eyeball in healthy volunteers [2]. Leptomeningeal failures of primary brain tumors in the optic nerves are rarely observed [3], [4], [5], [6]. This can be explained by the fact that optic nerves, even when not specifically targeted during CSI, usually received a therapeutic dose with the lateral opposed beams of the classic 3D-conventional photon technique. However, Rene et al. demonstrated that with modern intensity modulated radiation therapy (IMRT) planning techniques, there is a risk of underdosage of especially the anterior half of the optic nerves if not intentionally targeted, and that there is no margin for setup error regarding optic nerve dose coverage [7].

If the goal of CSI is to include entire optic nerves, adjacent eye-structures may more easily receive clinically detrimental radiotherapy doses. The lens is a radiosensitive structure, with radiotherapy-induced cataracts developing months to years after radiotherapy, and subject to a dose–response effect as well as an inverse dose-latency effect [8], [9], [10], [11]. A recent publication reported a threshold of 7 Gy in mean lens dose (MLD) to keep cataract risk below 25% [12]. At doses of 20–60 Gy, the risk increases to 35% within 5 years post-radiotherapy [8].

With widespread implementation of highly conformal planning techniques for CSI, individualized improvement of target volume coverage and sparing of organs-at-risk (OAR) is attainable. This forces reflection on potential pitfalls. Optic nerve target volumes and lenses are small structures and subject to intra- and inter-fraction movement. Eye movement may influence dose coverage, resulting in an increased risk of CSF recurrence and/or cataract development. Also for other tumors in the (peri-)orbital region in children and adults, eye movement may influence the dose distribution on target volumes and OAR, and information on amplitudes is valuable for daily practice of various radiotherapy subspecialties. For planning procedures within this study, we focused on the situation of CSI for high-risk medulloblastoma indications, where the lens would classify as OAR and the optic nerve as target volume.

The purpose of this study was to evaluate movement amplitudes of lenses and peripheral optic nerves in different gaze directions with MRI, and to evaluate the impact of eye movement on the CSI dose distribution on lens and orbital optic nerve (OON), for different planning techniques with photons and protons. Furthermore, the effect of using an internal target volume (ITV) around the optic disc and a planning organ-at-risk volume (PRV) around the lens was evaluated.

## 2 Materials and methods

### 2.1 Study population

Ten healthy adults, 2 men and 8 women aged between 21 and 51, volunteered to participate in this study. All candidates provided written consent before participation. Approval for the study was obtained from the University Medical Center Utrecht (UMCU) Research Ethics Committee (Protocol ID 15-466; ABR NL53099.041.15).

### 2.2 Scanning procedure

Between May and July 2019, volunteers underwent an MRI-scan (Philips, Ingenia, 1.5 Tesla) of the brain and skull-base in the radiotherapy department of the UMCU. The scans were generated in supine position on a soft mattress with knee support and the neck in neutral position. For imaging of the brain, a Philips dStream Headspine coil was used. To evaluate the position of the lenses, optic discs and optic nerves, T1-weighted images were obtained (3D T1 TFE acquisition, resolution 1 mm^3^ isotropic, FOV 230x230x160 mm, TE/TR = 3.5/7.7 ms, flip angle 8°) during 4 min and 31 s. On these images, the lenses, optic discs and optic nerves are rendered gray and surrounding tissue white. Five scans were made per volunteer; with the gaze direction in neutral position, and maximum left, right, cranial and caudal position (Supplementary Fig. 1).

### 2.3 Movement documentation

MRI’s of the different gaze directions were matched to the MRI in “neutral” eye position by a normalized mutual information algorithm [13], which was restricted to rigid registration according to the department’s clinical protocol. The clipbox for the registration, which was manually placed for each subject, included the entire skull. Quality of these image registrations is not hampered by scanning without a fixation mask [14]. On all MRI’s, delineations of different structures, among which lenses and optic discs, were performed by two experienced radiation oncologists (GJ, BH), using an in-house developed contouring software system [13]. The movement amplitude of the lens was determined by the coordinates of the center of mass (COM), while greatest movement amplitude of the moveable orbital part of the optic nerve was determined by the coordinates of the COM of the optic disc. Coordinates of the COMs were recorded for each gaze direction in all ten volunteers and per eye (left/right). The coordinates of the neutral position were used as reference for the movement amplitude in the different directions on an x, y, z grid, where x marks the left–right axis, y the anteroposterior axis and z the craniocaudal axis. Gaze deviation would displace the COMs along all axes. X-values decrease towards the subject’s right, y-values decrease towards the subject’s anterior and z-values decrease in the caudal direction (Supplementary Fig. 2).

### 2.4 Treatment delineation and planning

The “neutral” eye position scan was used for generation of radiotherapy plans. Several structures were delineated on this MRI:

The cranial clinical target volume (CTV) was delineated following the SIOPE recommendations [1].

As a separate substructure within the CTV, the cribriform plate was delineated to ensure that this risk area was not underdosed.

The optic nerves were delineated from the chiasm until and including the optic disc in the eye as part of the CTV in neutral gaze direction only. Additionally, the moving substructures “orbital optic nerves” (OON) and “optic discs” were delineated in all gaze directions.

As OARs, lenses and eyeballs were delineated in all gaze directions.

The CTV was expanded with a 3-mm isotropic margin to generate the planning target volume (PTV).

Trained convolutional neural networks were employed to generate synthetic CT’s (sCT) from MRI (Supplementary Fig. 1), as described previously [15]. The sCT’s were imported in the Monaco treatment planning system (version 5.11.02, Elekta AB, Stockholm, Sweden) of the UMCU for photon planning, and in Raystation 9A (Raysearch Laboratories AB, Sweden) of the University Medical Center Groningen (UMCG) for pencil-beam scanning (PBS) proton planning. The prescription dose was 36 Gy in 20 fractions, representing high-risk disease target dose. Volumetric Modulated Arc Therapy (VMAT) and PBS proton plans were constructed for all 10 subjects, while 3D-conventional photon plans were constructed only for subject 1 to 5 as proof-of-principle comparison of this classic technique with 2 highly conformal planning modalities.

Photon plans obtained a V95% >98% coverage to the PTV (including OON in “neutral” position and lamina cribrosa as separately reviewed targets), Dmax 110%, using a 2-mm grid size.

The 3D-conventional photon plans were created using 2 lateral 6-MV beams (gantry angles 90° and 270°±5°). VMAT plans consisted of a 6-MV FullArc.

For feasibility evaluation, adjusted VMAT plans were generated with an additional ITV_optic disc_ around the optic discs and a PRV_lens_ around the lenses, which were devised by using the 10 volunteers’ mean movement amplitudes + 1 Standard Deviation (SD) on al grid axes, per gaze direction.

Proton plans obtained a V95% >98% coverage to the voxelwise minimum CTV (CTV_voxelwise min_) (including OONs and lamina cribrosa as separately reviewed targets) [16], Dmax 110%, using a 2-mm grid size.

Proton plans consisted of two lateral oblique coplanar beam directions (gantry angles 110° and 250°) as typically used for whole brain irradiation at UMCG. Both equally weighted fields delivered uniform doses to the cranial CTV target volume under 3% beam range uncertainty and 3-mm isotropic patient setup uncertainty.

Dose coverage of target structures in neutral gaze position (cranial target volume, cribriform plate, OON) was evaluated as CTV_voxelwise min_ D98 for proton plans and nominal dose PTV D98 for photon plans, described as reliable criteria to compare coverage and robustness by Korevaar et al. [16]. Mean OAR dose for proton plans was evaluated in the nominal dose plan.

All delineated Regions Of Interest (ROIs) of the lenses and OONs in different gaze directions were projected on the “neutral” eye position dose plan, to evaluate dosimetric changes over these structures caused by eye movement.

### 2.5 Statistics

The coordinates on the x-,y-,z-grid of the COMs for the lenses and optic discs of each volunteer were documented in excel for each gaze direction. Position shifts were calculated in mm difference from the neutral position on all three grid axes. For each gaze direction the mean, median, SD, and range of position shifts were calculated.

For evaluation of dose distribution, the mean lens doses (MLD) and D98 of the OONs (D98_OON_) with the different planning techniques were registered in excel for each volunteer in each gaze direction. The mean, median, SD, minimum and maximum values of these dose parameters were calculated. For graphic display, MLD and D98_OON_ values were registered in Graphpad Prism® version 8.4.3 (GraphPad Software, San Diego, CA, USA).

We evaluated if adding a PRV_lens_ and ITV_optic disc_ during VMAT planning would significantly change MLD and D98_OON_ compared with the original VMAT plans. Shapiro-Wilk tests, normality QQ plots and Kolmogorov-Smirnov normality tests were performed with Graphpad Prism®, and afterwards paired t-tests were performed to analyze potential significant differences (p <0.05) in MLD and D98_OON_ over all gaze directions, between VMAT and VMAT with an added ITV_optic disc_ and PRV_lens_.

## 3 Results

### 3.1 Movement amplitudes of the lens and optic disc

Upon eye movement, the lens and optic disc COM shift along the x-, y- and z-axes. However, in the left–right and craniocaudal gaze deviations, COM shifts from neutral for both structures were largest along the x- and z-axis, respectively, for the 10 volunteers.

Table 1 demonstrates the mean shift from neutral position for lenses on these axes: left gaze 4.9±0.8 mm (± SD), and right gaze −5.2±1.1 mm on the x-axis. Cranial gaze 3.0±0.8 mm, and caudal gaze −5.8±1.2 mm on the z-axis.

**Table 1:** Movement amplitudes from neutral gaze (in mm) of 10 subjects’ lenses and optic discs, for different gaze directions along the left–right x-axis and craniocaudal z-axis. In the x-,y-,z-grid of the MRI, x-values decrease towards the subject’s right, and z-values decrease in the caudal direction. Mean±SD, minimum (lowest value) and maximum (highest value) shifts are given. ROI = Region Of Interest.

ROI	Left gaze;shift x (mm)	Right gaze;shift x (mm)	Cranial gaze; shift z (mm)	Caudal gaze; shift z (mm)
Lenses				
Minimum	3.3	−6.8	1.9	−9.1
Maximum	6.7	−3.3	4.2	−3.1
MEAN LENSES±SD	4.9±0.8	−5.2±1.1	3.0±0.8	−5.8±1.2
Optic discs				
Minimum	−6.8	2.6	−8.3	2.5
Maximum	−3.4	7.1	−1.6	9.8
MEAN OPTIC DISCS±SD	−5.0±1.0	5.5±1.2	−4.2±2.0	7.0±2.0

For optic discs, mean shift from neutral position on these axes were: left gaze −5.0±1.0 mm, and right gaze 5.5±1.2 mm on the x-axis. Cranial gaze −4.2±2.0 mm, and caudal gaze 7.0±2.0 mm on the z-axis.

COM shifts along all 3 axes are given in Supplementary Table 1.

### 3.2 Target volume coverage in neutral gaze direction

**Fig. 1:**
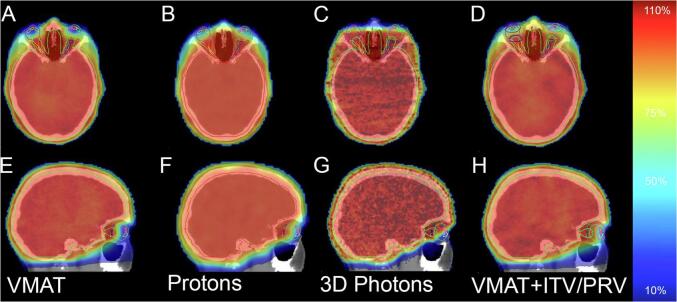
Examples of ROI structures and 36-Gy target dose distribution as planned with the eyes in neutral gaze direction for the different planning techniques, in axial (A–D) and sagittal (E-H) view. Eyeball ROIs are depicted in the neutral gaze position. All images show the same ROIs of lenses and orbital optic nerves in multiple gaze directions. (A + E) Dose distribution on PTV (delineated in green) with VMAT. (B + F) Dose distribution on CTV (delineated in red) with PBS protons in the CTV_voxelwise min_ plan. (C + G) Dose distribution on PTV (delineated in green) with 3D-conventional opposing photon beams. (D + H) Dose distribution on PTV (delineated in green) with VMAT, where an ITV for optic discs (blue / green) and PRV for lenses (red / purple) were added in the planning process. Colormap with relative dose values on the right. (For interpretation of the references to color in this figure legend, the reader is referred to the web version of this article.)

For the different planning techniques, target volume coverage goals for the entire cranial volume, as well as the encompassed sub-target volumes of the cribriform plates and OONs, were met in all subjects. The mean D98 target volumes coverage for PTV in photon plans and CTV_voxelwise min_ in proton plans is given in Supplementary Table 2. Fig. 1 shows examples of ROI structures and dose distribution for the different planning techniques.

### 3.3 Impact of eye movement on the lens and orbital optic nerve dose

**Fig. 2:**
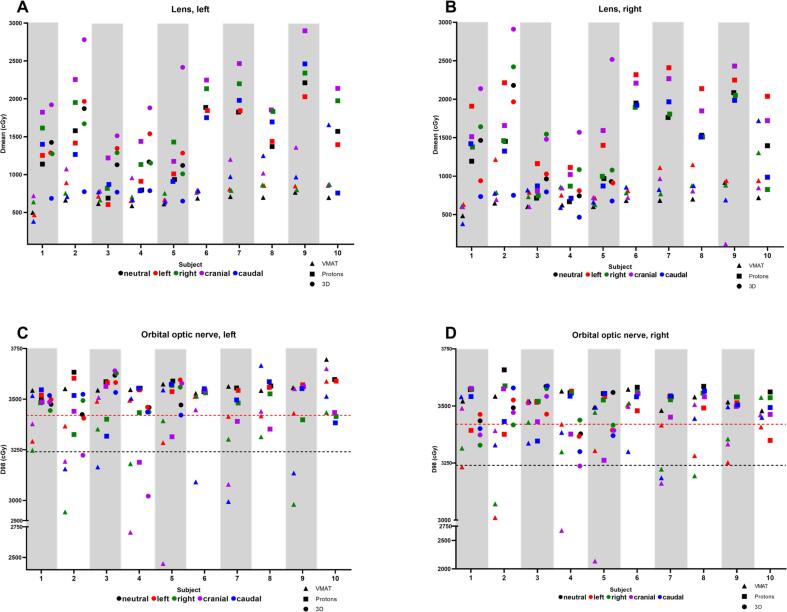
ROI dose deviations per subject for different gaze directions (neutral (black); left (red); right (green); cranial (purple); caudal (blue)) when a radiotherapy plan was constructed with the eyes in neutral gaze, using different planning techniques (VMAT, PBS protons, 3D-conventional photons). Dmean is given in cGy for the left lens (A) and the right lens (B). D98 is given in cGy for the left orbital optic nerve (C) and right orbital optic nerve (D). In (C) and (D), the black and red dotted lines in the graph represent 90% and 95% of the prescribed 36-Gy dose, respectively. (For interpretation of the references to color in this figure legend, the reader is referred to the web version of this article.)

After planning in the neutral gaze direction, a caudal gaze direction in 3D-conventional plans decreased the left and right MLD with mean −6.1 Gy and −5.7 Gy (-16.9% and -15.8% relative to prescription dose), respectively, while hardly affecting the mean D98_OON_ (-0.02 Gy and 0.4 Gy (-0.06% and 1.1% relative to prescription dose), respectively; Table 2, Fig. 2).

**Table 2:** Dose differences in the four evaluated gaze directions, compared to the dose in neutral gaze, for VMAT photon plans, PBS proton plans and 3D-conventional photon plans. Dose differences are given in Mean [range] of the mean dose to the lenses (MLD) and Mean [range] of the D98 of the orbital optic nerves (D98_OON_). *5 3D-conventional photon plans were evaluated versus 10 for the other techniques.

Gaze direction	Dose difference from Neutral gaze; Mean MLD Gy [range]					
	VMAT		Proton		Conventional*	
	Left lens	Right Lens	Left lens	Right Lens	Left lens	Right lens
Left	1.0 [−0.3 to 2.3]	2.4 [0.2 to 5.7]	−0.3 [−1.8 to 1.2]	5.2 [−1.6 to 7.6]	1.4 [−1.4 to 3.7]	−1.3 [−5.3 to 0.7]
Right	2.9 [0.5 to 5.9]	1.6 [−0.3 to 2.8]	5.5 [2.4 to 6.9]	−0.2 [−5.7 to 2.0]	−0.6 [−2.0 to 1.6]	3.0 [1.5 to 5.8]
Cranial	1.1 [0.3 to 1.7]	0.3 [−8.0 to 2.8]	3.4 [1.3 to 5.0]	3.3 [0.9 to 6.2]	7.6 [3.8 to 13.0]	8.7 [5.2 to 15.9]
Caudal	2.3 [−1.2 to 9.6]	1.5 [−2.3 to 10]	−0.1 [−8.1 to 3.3]	−0.2 [−4.1 to 2.3]	−6.1 [−11.0 to −3.6]	−5.7 [−14.3 to −1.7]

	Dose difference from Neutral gaze; Mean D98_OON_ Gy [range]					
	VMAT		Proton		Conventional*	
	Left optic nerve	Right optic nerve	Left optic nerve	Right optic nerve	Left optic nerve	Right optic nerve
Left	−1.4 [−2.9 to −0.2]	−1.8 [−5.3 to 0]	−1.4 [−3.0 to −0.09]	−2.2 [−6.5 to 0.01]	0.2 [−0.4 to 1.2]	−0.5 [−1.7 to 0.3]
Right	−3.0 [−6.1 to −0.1]	−1.8 [−4.7 to 0.6]	−1.1 [−3.1 to −0.06]	−0.2 [−0.7 to 0.0]	0.2 [−0.3 to 0.9]	−0.6 [−1.4 to 0.6]
Cranial	−3.2 [−11.2 to −0.06]	−3.3 [−13.6 to −0.07]	−1.4 [−3.7 to 0.05]	−1.0 [−2.9 to −0.3]	−1.0 [−4.3 to 1.1]	−0.9 [−1.7 to −0.2]
Caudal	−2.4 [−5.7 to 1.2]	−1.4 [−3.0 to 0.0]	−0.6 [−2.7 to 0.4]	−0.6 [−2.3 to 0.04]	−0.02 [−0.9 to 1]	0.4 [−1.9 to 0.9]

For VMAT and protons plans, changing to other-than-neutral gaze directions mainly increased the MLD and decreased the D98_OON_ (Table 2, Fig. 2). However, wide and non-uniform dose-difference ranges were observed over the various gaze directions (MLD -8.1 Gy to 10 Gy (–22.5% to 27.8% relative to prescription dose); D98_OON_ -13.6 Gy to 1.2 Gy (-37.8% to 3.3% relative to prescription dose) (Table 2).

For the 10 subjects combined, with a mean D98_OON_ decrease up to 3.3 Gy (9.2%), the mean D98_OON_ decrease was <10% of the prescribed 36-Gy dose for all 3 planning techniques (Table 2). For all registered D98_OON_ doses of the 3 planning techniques in 5 gaze directions, the D98_OON_ dose was <90% of the prescribed 36-Gy dose (<32.4 Gy) in 25 of 250 dose-registrations (Fig. 2C/D).

### 3.4 Dose impact of a lens PRV and an optic disc ITV

**Fig. 3:**
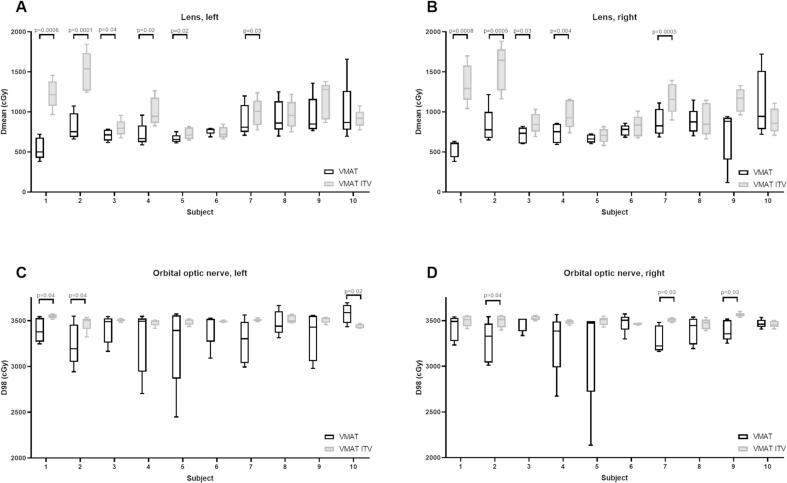
ROI dose differences in all 5 gaze directions per subject for the mean doses on the left lens (A) and the right lens (B); and for the D98 doses on the left orbital optic nerve (C) and right orbital optic nerve (D), for the standard VMAT plan (VMAT) compared with the VMAT plan with integrated PRV for lenses and ITV for orbital optic discs (VMAT ITV). Box-and-whisker plots are given for the dose range in cGy for all gaze directions per subject. Boxes: median value and upper and lower quartiles; whiskers: minimum and maximum data point. Paired *t*-test significant differences (p <0.05) between the two planning techniques are marked, with the corresponding p-values.

PRV_lens_ dimensions of 4, 7, 6, 7, 1, and 4 mm; and ITV_optic disc_ dimensions of 9, 7, 7, 6, 5, and 1 mm in cranial, caudal, left, right, anterior and posterior direction, respectively, were derived from mean + 1 SD COM shifts of the lenses and the optic discs from neutral position. An ITV_optic disc_ coverage of V95% >98% was reached for all subjects except volunteers 5 and 9 (V95% >96%). D98_OON_ was ≥34.2 Gy (95% of prescribed dose) in 95/100 evaluated gaze directions, and ≥32.4 Gy (90% of prescribed dose) in 100/100 evaluated gaze directions, compared to respectively 57/100 and 79/100 measurements in the original VMAT plans (Figs. 2,  3C/D). Compared with the original VMAT plan, steering on ITV_optic disc_ and PRV_lens_ significantly increased MLD over all gaze directions to one or both lenses for 6/10 and 5/10 subjects, respectively (Fig. 3A/B). It significantly changed D98_OON_ over all gaze directions of one or both OONs in 5/10 and 1/10 subjects, respectively (Fig. 3C/D).

## 4 Discussion

In this study we evaluated MRI-documented eye structure movements, and influence of eye movements on lens dose and optic nerve target coverage in CSI plans. The outcomes of eye movements and examples of influence on dose coverage with different planning techniques may help readers to form conclusions for their own clinic’s cranial radiotherapy setup. Our findings also provide practical information regarding OAR and target volume movement amplitudes applicable to various pediatric and adult indications for (peri-)orbital radiotherapy, such as sarcomas, head-and-neck tumors, central nervous system tumors and hematological malignancies.

MRI is a validated method to explore movement of the separate eye components [17], [18], [19]. For lenses and optic discs, the latter considered as the most peripheral part of the optic nerve, mean movement amplitudes of all 10 volunteers in our study ranged between±3 mm to±7 mm, with overall largest amplitudes, of even > 9 mm, in the caudal gaze direction. This is comparable to eye movement studies, where largest displacement angles were measured with caudal gaze [20], [21]. Ocular biometry- or MRI-based studies found that during gaze shifts, the axial length of the eyeball changes slightly - with largest elongation in downward gaze [22], and the posterior segment of the eye shows more displacement from the anterior segment during vertical than during horizontal displacements [18]. Hence, the eyeball reshapes during movement. This is in line with our results, where we found that shifts of lenses were comparable, but not exactly opposite to shifts of optic discs.

Eye movement of the ten volunteers influenced dose on lenses and orbital optic nerves, when CSI plans were constructed with neutral gaze direction using three different planning techniques. For VMAT and proton therapy, the optimal dose distribution on lenses and optical nerves was mainly in the planned-on neutral gaze. For the 3D-conventional photon plans, caudal gaze direction produced the lowest MLDs, which concurs with the traditional advice to look downward during conventional whole-brain radiotherapy. The mostly favorable orbital optic nerve target coverage over all gaze directions in this study supports previous findings that with this classic technique, tumor failures in the optical nerve leptomeningeal extension were rare. For all planning techniques combined, the mean orbital optic nerve D98 decrease for different gaze directions was between 0.02 Gy and 3.3 Gy. D98_OON_ decreased to > 10% of the prescribed dose in 25 of the 250 eye position registrations. As this occurred mainly with standard VMAT plans, which displayed optimal coverage of “neutral” optic nerve position and overall lower lens doses than proton plans, this is a notion to be taken into consideration; constraining the lens dose may infringe on optic nerve target volume coverage in case of eye movement.

When inter- and intra-fraction eye movements occur during the course of a CSI treatment schedule, directions and amplitudes will be variable, and dose deviations will be different from the extreme “static deviation” analyses reported in this study. However, as we have shown with conformal planning modalities, eye movement results in uni- or bilateral suboptimal organ sparing of lenses and target coverage of optic nerves in all gaze directions. If one wishes to reduce the dose-variations, several options are at hand. Gaze fixation can be achieved with the use of static fixtures to the positioning set-up, with the patient focusing on a specific point. An easy implementable setup is e.g. using a bended plexiglas strip that is stably attached to the mask or head rest and contains a single marker to fixate on during scanning and treatment. It is also possible to include a tracking system for high-precision treatment [23], [24]. For 3D-conventional lateral-beam photon plans, fixating the gaze downward is optimal for lens sparing. For other modalities, the neutral gaze is most relaxed and can be accounted for in the planning process. If desired by a patient, the eye position with relaxed closed eyes can be stable, but this should be evaluated with e.g. conebeam-CT during treatment, and planning dose calculations should include evaluation of a potential “bolus” effect of the eyelids over the lenses.

Gaze fixation will usually not be an option for very young children and most children irradiated under sedation, depending on the type and depth of sedation. In those cases, the use of an ITV for orbital optic nerve volumes or a PRV for lens volumes could be applied. As we demonstrated, implementing both an ITV_optic disc_ and a PRV_lens_ in the VMAT plan will result in a higher mean lens dose. Adding an ITV_optic disc_ produced a significant change in the bilateral orbital optic nerve D98 over all gaze directions in only one subject. However, D98 ranges became much narrower and above 90–95% of the prescribed dose, resulting in more robustness compared with the original VMAT plans. Therefore, addition of an ITV does preclude underdosage risk of moving optic nerve target volumes, but may be of less consequence when eye deviation is small and variable over all fractions. When resorting to such safety volumes, it is at the judgment of the treating physician which takes precedence. Based on adult volunteers, a PRV diameter of CC 14 (x LR 14 × AP 8) mm for the lens, and an ITV diameter of CC 18 (x LR 14 × AP 10) mm for the optic disc cover most eye movements to peripheral positions. These dimensions may be useful in radiotherapy planning for other (peri-)orbital tumors as well. PRV_lens_ may be decreased or disregarded when optimal optic nerve coverage is vital. During treatment, eye movements will be less extreme than registered here, and smaller PRV_lenses_ may be applicable. Further study in patients is recommended to evaluate this, in which natural eye movements may be captured comfortably with fast-MRI instead of static MRI [19].

A pitfall of the study, regarding extrapolation to pediatric cases, is that all evaluations were performed in adult subjects. Especially in young children, the frontal sinus is still in development, and the position of the eye in the developing orbit is closer to the CSI target volume. The eye itself grows from a diameter of approximately 16–17 mm at birth to its final size of 24 mm around age 7–8 years [25]. Ninety-five percent of orbital growth is finished at age 11 for girls and 15 for boys [26]. Eye movement amplitudes of especially post-toddler aged children may therefore not differ greatly from the observed ranges in this study.

In conclusion, eye movements mainly increase lens doses for conformal planned CSI, while the mean detriment for orbital optic nerve D98 coverage is <10% of the prescribed dose.

## Declaration of Competing Interest

The authors declare that they have no known competing financial interests or personal relationships that could have appeared to influence the work reported in this paper.
