# Supplementary material for: Influence of eye movement on lens dose and optic nerve target coverage during craniospinal irradiation
Source: Clin Transl Radiat Oncol. 2021 Aug 29;31:28–33. doi: 10.1016/j.ctro.2021.08.009 (PMC8427085; doi:10.1016/j.ctro.2021.08.009)
Supplement: Supplementary data 1 [file mmc1.pdf]

ROI and volunteer no.	Left gaze; Shift X	Left gaze; Shift Y	Left gaze; Shift Z	Right gaze; Shift X	Right gaze; Shift Y	Right gaze; Shift Z	Cranial gaze; Shift X	Cranial gaze; Shift Y	Cranial gaze; Shift Z	Caudal gaze; Shift X	Caudal gaze; Shift Y	Caudal gaze; Shift Z
Lens Left												
Subject												
1	5.32	4.63	-1.87	-6.40	1.33	-0.71	-1.27	-0.18	1.63	-1.51	5.31	-4.25
2	5.11	2.75	-1.89	-6.07	1.83	-2.42	-1.03	0.74	2.60	-0.54	0.85	-6.07
3	3.90	1.28	-0.57	-5.36	1.20	-0.88	-0.40	0.81	2.43	-1.20	1.35	-5.34
4	4.00	1.98	-0.10	-6.80	1.80	-1.13	-1.04	0.27	3.70	-1.81	1.69	-5.18
5	4.67	2.74	-2.00	-3.51	1.63	-1.99	-0.54	1.61	3.85	-0.91	1.83	-4.72
6	4.16	1.32	-0.11	-4.29	0.78	-0.82	0.54	0.89	2.53	-0.64	1.44	-5.96
7	5.37	2.58	0.83	-5.35	2.01	-1.81	-1.02	1.62	3.28	-2.07	2.21	-6.61
8	4.80	2.82	-1.18	-5.95	1.56	-1.14	-0.90	1.26	3.20	-4.66	4.25	-6.68
9	3.30	2.49	-4.72	-5.52	3.07	-3.22	-0.59	0.27	1.26	-1.07	2.86	-6.05
10	4.87	1.94	-1.23	-6.21	1.74	-1.56	-1.17	1.38	3.58	-0.23	2.11	-5.87
Mean	4.55	2.45	-1.28	-5.55	1.70	-1.57	-0.74	0.87	2.81	-1.46	2.39	-5.67
Median	4.74	2.54	-1.21	-5.74	1.69	-1.35	-0.96	0.85	2.90	-1.14	1.97	-5.92
Minimum	3.30	1.28	-4.72	-6.80	0.78	-3.22	-1.27	-0.18	1.26	-4.66	0.85	-6.68
Maximum	5.37	4.63	0.83	-3.51	3.07	-0.71	0.54	1.62	3.85	-0.23	5.31	-4.25
Lens Right												
Subject												
1	6.68	3.89	-1.31	-5.87	3.01	0.94	-0.22	-1.05	2.55	1.06	5.40	-3.05
2	5.98	1.63	-2.47	-5.53	3.12	-1.87	-0.75	1.06	2.60	0.47	1.22	-6.69
3	4.74	0.61	-0.96	-4.36	2.26	-0.66	0.09	0.98	3.21	0.65	1.35	-5.38
4	4.37	0.87	0.12	-6.42	3.17	-0.39	-0.80	0.55	4.09	-0.53	2.38	-5.51
5	4.98	1.96	-2.70	-3.30	2.18	-1.71	0.21	1.68	4.18	-0.27	1.73	-4.57
6	5.18	0.20	-0.93	-3.28	1.51	-0.40	1.51	0.62	2.05	1.60	1.66	-6.42
7	5.61	1.62	-0.46	-5.21	2.91	-0.98	0.14	2.00	3.78	3.43	2.18	-6.01
8	5.30	1.81	-1.69	-5.92	3.19	-1.50	0.04	1.25	3.03	1.69	4.72	-9.08
9	4.32	1.17	-3.57	-4.70	3.44	-2.59	1.69	0.18	1.93	0.79	2.16	-5.67
10	5.95	1.71	-1.27	-4.23	1.82	-1.00	-0.80	1.87	3.74	2.93	2.45	-6.37
Mean	5.31	1.55	-1.53	-4.88	2.66	-1.02	0.11	0.91	3.12	1.18	2.52	-5.88
Median	5.24	1.63	-1.29	-4.96	2.96	-0.99	0.07	1.02	3.12	0.92	2.17	-5.84
Minimum	4.32	0.20	-3.57	-6.42	1.51	-2.59	-0.80	-1.05	1.93	-0.53	1.22	-9.08
Maximum	6.68	3.89	0.12	-3.28	3.44	0.94	1.69	2.00	4.18	3.43	5.40	-3.05
**MEAN LENSES**	**4.93**			**-5.21**					**2.96**			**-5.77**
OD Left												
Subject												
1	-3.93	-6.45	2.46	6.72	-1.09	1.96	-1.84	0.08	-1.63	-1.53	-6.60	6.53
2	-4.84	-3.12	1.64	7.08	-0.21	2.32	1.10	-0.99	-4.11	-0.75	-3.20	7.62
3	-4.39	-2.50	0.76	6.43	-0.56	1.09	-0.48	-1.33	-2.97	-0.35	-1.32	7.33
4	-3.79	-2.83	-1.20	6.91	-0.06	0.05	-0.22	-1.55	-6.83	-0.65	-2.31	4.51
5	-3.51	-3.42	0.51	3.90	0.24	1.01	1.01	-0.84	-7.51	0.53	-1.70	2.83
6	-4.91	-2.83	0.65	5.10	-0.19	0.38	-1.01	-1.23	-2.93	-1.69	-2.86	8.47
7	-4.46	-3.21	-0.25	6.79	0.33	0.90	0.71	-1.40	-5.90	-0.89	-2.55	7.15
8	-5.33	-3.38	1.10	5.52	0.84	1.26	0.33	-0.82	-3.88	-1.18	-2.11	9.37
9	-3.39	-2.85	4.90	5.12	-0.72	5.45	-0.09	-0.02	-2.19	-0.90	-3.20	8.24
10	-4.50	-2.44	2.24	7.06	0.51	2.41	0.44	-1.26	-3.86	-1.46	-2.37	9.18
Mean	-4.31	-3.30	1.28	6.06	-0.09	1.68	0.00	-0.94	-4.18	-0.89	-2.82	7.12

Median	-4.43	-2.99	0.93	6.58	-0.12	1.18	0.12	-1.11	-3.87	-0.90	-2.46	7.48
Minimum	-5.33	-6.45	-1.20	3.90	-1.09	0.05	-1.84	-1.55	-7.51	-1.69	-6.60	2.83
Maximum	-3.39	-2.44	4.90	7.08	0.84	5.45	1.10	0.08	-1.63	0.53	-1.32	9.37
OD Right												
Subject												
1	-6.50	-3.74	3.53	4.90	-4.10	-0.28	-0.93	0.43	-2.25	0.10	-6.82	7.70
2	-6.13	-0.05	2.64	6.04	-6.39	2.34	1.35	-1.54	-3.30	0.06	-2.68	7.56
3	-4.86	0.58	0.46	4.82	-3.87	0.57	0.00	-1.01	-2.39	0.09	-1.03	6.71
4	-4.39	0.15	-1.52	4.33	-2.95	-0.61	1.57	-1.66	-6.83	1.32	-3.58	4.55
5	-5.52	0.00	1.06	2.61	-1.45	0.13	-1.47	-1.39	-8.31	0.01	-1.44	2.51
6	-5.65	0.52	0.70	4.64	-2.88	0.61	-1.14	0.17	-3.14	0.11	-1.92	8.22
7	-6.37	0.69	-0.64	6.16	-5.52	0.87	0.90	-1.54	-5.55	-1.02	-2.69	7.56
8	-5.88	0.05	1.20	6.22	-6.13	0.31	0.68	-1.39	-4.36	-0.20	-1.47	6.40
9	-5.07	-0.23	4.42	5.08	-4.95	3.01	-1.42	0.03	-3.76	0.44	-3.31	7.83
10	-6.78	0.64	2.34	4.63	-2.04	2.51	1.51	-1.91	-2.62	-0.20	-1.51	9.77
Mean	-5.71	-0.14	1.42	4.94	-4.03	0.95	0.11	-0.98	-4.25	0.07	-2.64	6.88
Median	-5.76	0.10	1.13	4.86	-3.98	0.59	0.34	-1.39	-3.53	0.08	-2.30	7.56
Minimum	-6.78	-3.74	-1.52	2.61	-6.39	-0.61	-1.47	-1.91	-8.31	-1.02	-6.82	2.51
Maximum	-4.39	0.69	4.42	6.22	-1.45	3.01	1.57	0.43	-2.25	1.32	-1.03	9.77
**MEAN ODs**	**-5.01**			**5.50**					**-4.22**			**7.00**

Supplementary Table 1. Shifts from neutral gaze (in mm) of the center of mass (COM) of lenses and optic discs for different gaze directions, on an x- (left-right), y- (anterior-posterior) and z-grid (cranial-caudal), as measured in 10 subjects. In the x-,y-,z-grid of the MRI, x-values decrease towards the subject's right, y-values decrease towards the subject's anterior and z-values decrease in the caudal direction. Mean, median, minimum (lowest value) and maximum (highest value) shifts are given per left / right ROI in each gaze direction. Mean values of all lenses and optic discs combined are given for the most prominent movement amplitudes; in the x-direction for left and right gaze and in the z-direction for cranial and caudal gaze. OD = optic disc.
