# Supplementary material for: Influence of eye movement on lens dose and optic nerve target coverage during craniospinal irradiation
Source: Clin Transl Radiat Oncol. 2021 Aug 29;31:28–33. doi: 10.1016/j.ctro.2021.08.009 (PMC8427085; doi:10.1016/j.ctro.2021.08.009)
Supplement: Supplementary data 2 [file mmc2.pdf]

**Coverage; D98**	**VMAT**<br>Mean ±SD (Gy)	**VMAT with ITV / PRV**<br>Mean ±SD (Gy)	**Proton**<br>Mean ±SD (Gy)	**3D-Conventional***<br>Mean ±SD (Gy)
Entire cranial volume	34.4 ±0.3	34.1 ±0.2	34.9 ±0.06	33.7 ±0.4
Cribriform plate	34.7 ±0.4	34.2 ±0.5	34.7 ±0.3	33.9 ±1.0
Orbital optic nerve left	34.6 ±1.0	34.8 ±0.2	33.7 ±0.7	34.5 ±0.7
Orbital optic nerve right	34.6 ±0.4	34.7 ±0.2	33.5 ±0.8	34.1 ±0.8

Supplementary Table 2. Target coverage; mean D98 ±SD of subjects, using different planning techniques (VMAT, VMAT with inclusion of $ITV_{optic\ disc}$ and PRV_{lens}, PBS proton, 3D-conventional photon*), planned with the eyes in neutral gaze direction. Coverage is evaluated as D98 of the CTV in the voxelwise min plans for proton planning, and D98 of the PTV for photon plans. *5 3D-conventional photon plans were evaluated; 10 for the other techniques.
