# Supplementary material for: Influence of eye movement on lens dose and optic nerve target coverage during craniospinal irradiation
Source: Clin Transl Radiat Oncol. 2021 Aug 29;31:28–33. doi: 10.1016/j.ctro.2021.08.009 (PMC8427085; doi:10.1016/j.ctro.2021.08.009)
Supplement: Supplementary data 3 [file mmc3.pdf]

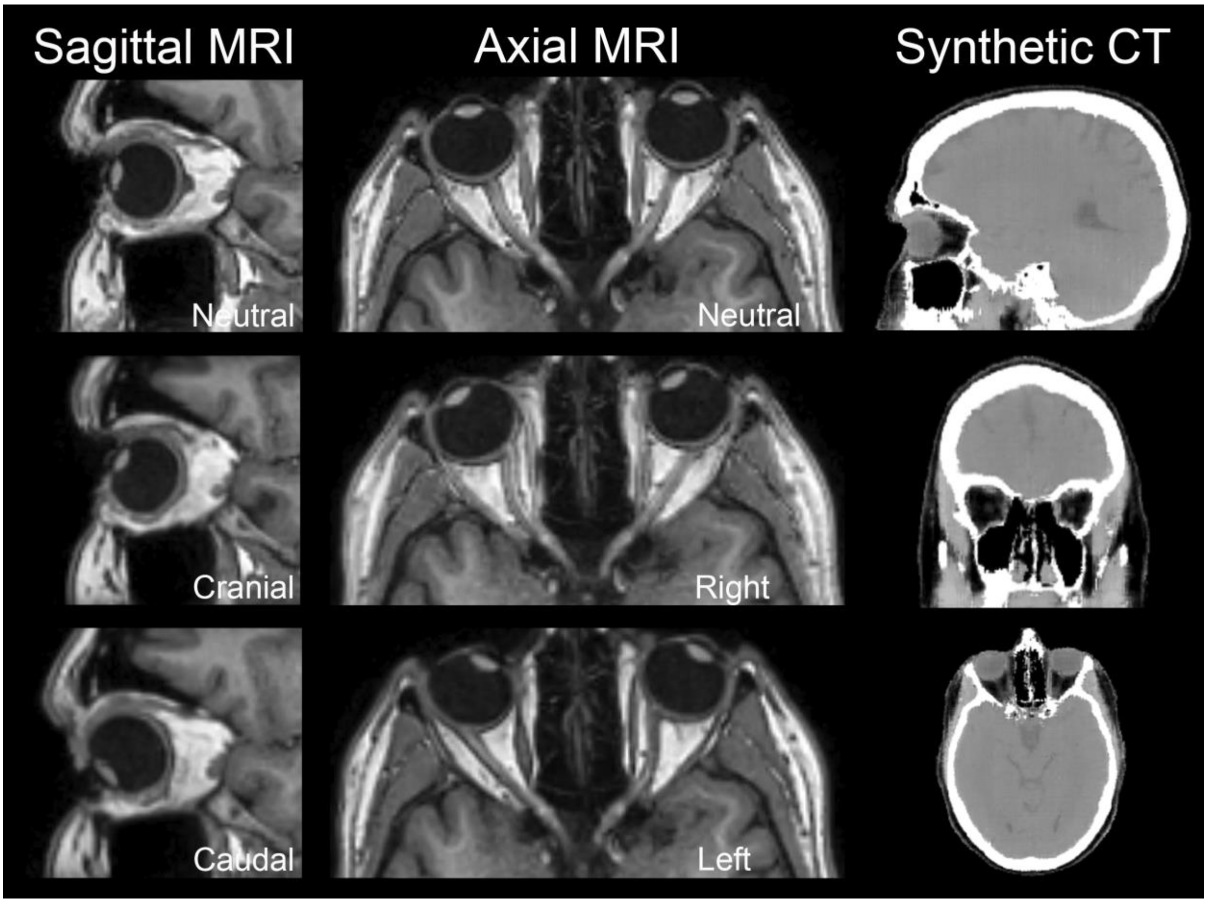

Supplementary Figure 1. T1-weighted MRI images of optic nerve and/or lens positions of a volunteer in sagittal and axial view, with gaze direction in neutral, cranial, caudal, left and right position. Synthetic CT images of the same subject in sagittal, coronal and axial view.
