# Supplementary material for: Influence of eye movement on lens dose and optic nerve target coverage during craniospinal irradiation
Source: Clin Transl Radiat Oncol. 2021 Aug 29;31:28–33. doi: 10.1016/j.ctro.2021.08.009 (PMC8427085; doi:10.1016/j.ctro.2021.08.009)
Supplement: Supplementary data 4 [file mmc4.pdf]

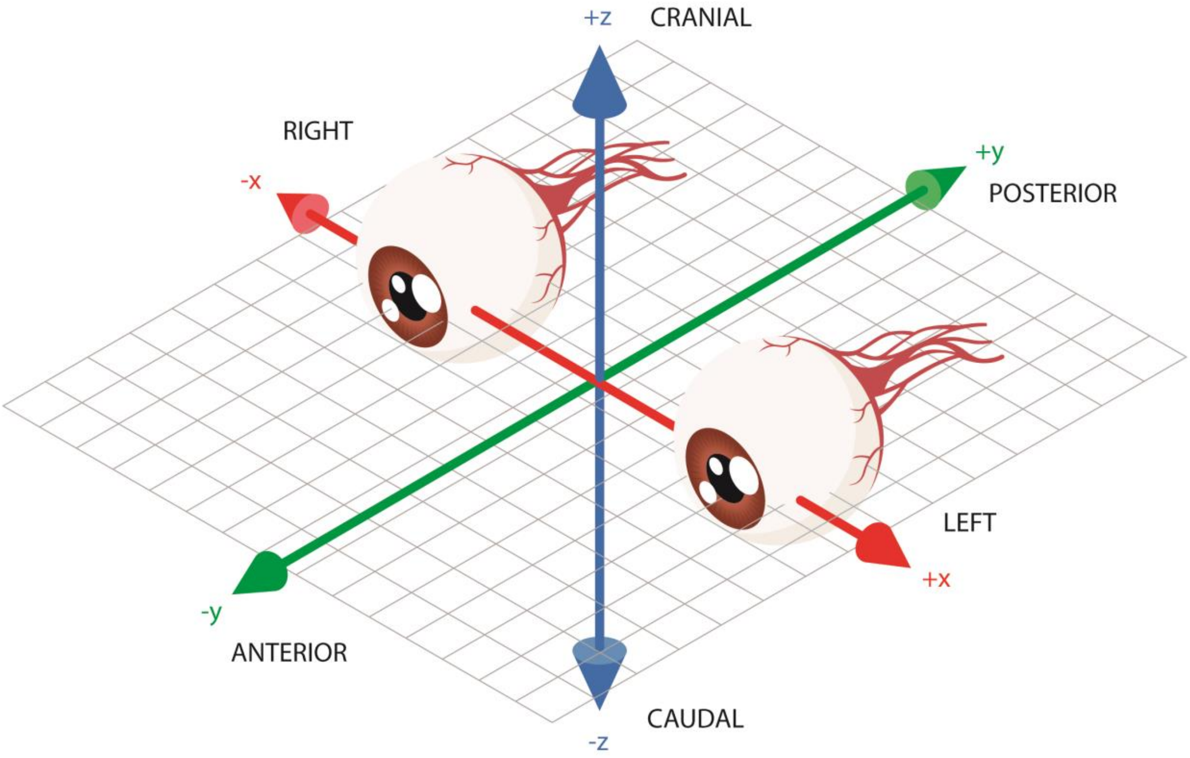

Supplementary Figure 2. Graphic representation of the MRI grid and orientation of the different gaze directions (here: neutral gaze direction). In the x-,y-,z-grid of the MRI, x-values decrease towards the subject's right, y-values decrease towards the subject's anterior and z-values decrease in the caudal direction. *Graphic design by C. van Kesteren, UMCU.*
